# A Review of Cascaded Metasurfaces for Advanced Integrated Devices

**DOI:** 10.3390/mi15121482

**Published:** 2024-12-10

**Authors:** Lingyun Zhang, Zeyu Zhao, Leying Tao, Yixiao Wang, Chi Zhang, Jianing Yang, Yongqiang Jiang, Huiqi Duan, Xiaoguang Zhao, Shaolong Chen, Zilun Wang

**Affiliations:** 1 State Key Laboratory of Precision Measuring Technology & Instruments, Tianjin University, Tianjin 300072, China; zhangly0319@outlook.com (L.Z.); zzykiwi@tju.edu.cn (Z.Z.); taoleying@tju.edu.cn (L.T.); yixiaowang@tju.edu.cn (Y.W.); 2 Department of Precision Instrument, Tsinghua University, Beijing 100084, China; 2421781108zc@gmail.com (C.Z.); 99yjn@163.com (J.Y.); zhaoxg@mail.tsinghua.edu.cn (X.Z.); 3 State Key Laboratory of Precision Measurement Technology and Instrument, Tsinghua University, Beijing 100084, China; 4 Beijing Advanced Innovation Center for Integrated Circuits, Tsinghua University, Beijing 100084, China; 5 State Key Laboratory of Pathogen and Biosecurity, Academy of Military Medical Sciences, Beijing 100071, China; jiangyq@bmi.ac.cn (Y.J.); d1449009136@163.com (H.D.)

**Keywords:** metamaterials, cascaded metasurfaces, tunable metasurfaces

## Abstract

This paper reviews the field of cascaded metasurfaces, which are advanced optical devices formed by stacking or serially arranging multiple metasurface layers. These structures leverage near-field and far-field electromagnetic (EM) coupling mechanisms to enhance functionalities beyond single-layer metasurfaces. This review comprehensively discusses the physical principles, design methodologies, and applications of cascaded metasurfaces, focusing on both static and dynamic configurations. Near-field-coupled structures create new resonant modes through strong EM interactions, allowing for efficient control of light properties like phase, polarization, and wave propagation. Far-field coupling, achieved through greater interlayer spacing, enables traditional optical methods for design, expanding applications to aberration correction, spectrometers, and retroreflectors. Dynamic configurations include tunable devices that adjust their optical characteristics through mechanical motion, making them valuable for applications in beam steering, varifocal lenses, and holography. This paper concludes with insights into the potential of cascaded metasurfaces to create multifunctional, compact optical systems, setting the stage for future innovations in miniaturized and integrated optical devices.

## 1. Introduction

After the advent of EM (electromagnetic) science, extensive investigations have been conducted on the EM properties of natural materials. In the 1960s, researchers began contemplating the availability of certain absent EM properties, such as simultaneously negative permittivity (*ε*) and permeability (*μ*), commonly referred to as left-handed materials [1]. After about 30 years, the demonstration of metamaterials solved this problem by periodically positioning artificially designed unit cells in 3D (three-dimensional) space [2]. While these unit cells are made from naturally occurring materials, their topology, geometry, and subsequent EM resonance play a pivotal role in determining the effective EM properties. The subwavelength sizes of the unit cells and their arrangement periodicity prevent high-order diffraction effects, enhance the spatial resolution of effective EM properties, and give rise to the name “meta-atoms” for unit cells. The concept of a metamaterial-based perfect lens gained a lot of attention [3]. Then, metamaterials with spatially varying unit cells were proposed, leading to gradient EM properties [4] and novel transformation optics [5], enabling applications including invisible cloaking [6], Luneburg lens [7], EM field concentrator [8], and so forth. However, the 3D metamaterials face fabrication challenges when extended to small working wavelengths.

As a 2D form of metamaterials, metasurfaces were first introduced through the generalized Snell’s law in 2011 [9,10]. These proposed metasurfaces are compatible with semiconductor fabrication technologies, facilitating batch production and commercial applications while enabling wavefront shaping in optical bands. The concept of metasurfaces incorporates subwavelength unit cells arranged in a designed order on a 2D plane, forming an ultrathin planar structure capable of manipulating the amplitude, phase, polarization state, nonlinear response, and orbital angular momentum (OAM) of EM waves at a subwavelength resolution [11]. These subwavelength unit cells allow enhanced light–matter interactions and impart abrupt changes in field parameters on the surface, thereby bypassing the traditional dependence on optical path length for conventional optical elements. This unprecedented ability of flexible light manipulation within the subwavelength thickness opens up avenues for miniaturized and integrated metadevices (metasurface devices [12]) for enhanced light field sensing [13,14,15,16,17] and beam shaping [18,19,20] applications. For instance, by incorporating periodically placed phase- and anisotropy-varying unit cells, metasurfaces enable multi-dimensional light field imaging applications with metalenses (metasurface lenses) [21,22,23,24,25] as well as multi-channel holography [26,27,28], precise metrology [29,30,31], and optical encryption [32,33]. Metasurfaces with periodic unit cells enable highly efficient absorbers [34,35] and nonlinear effects [36]. Moreover, nonlocal metasurfaces, which consider the perturbatively varying unit cells, exhibit promising potential in optical analog computing [37], spatial compression [38,39], and image processing [40].

**Figure 1:**
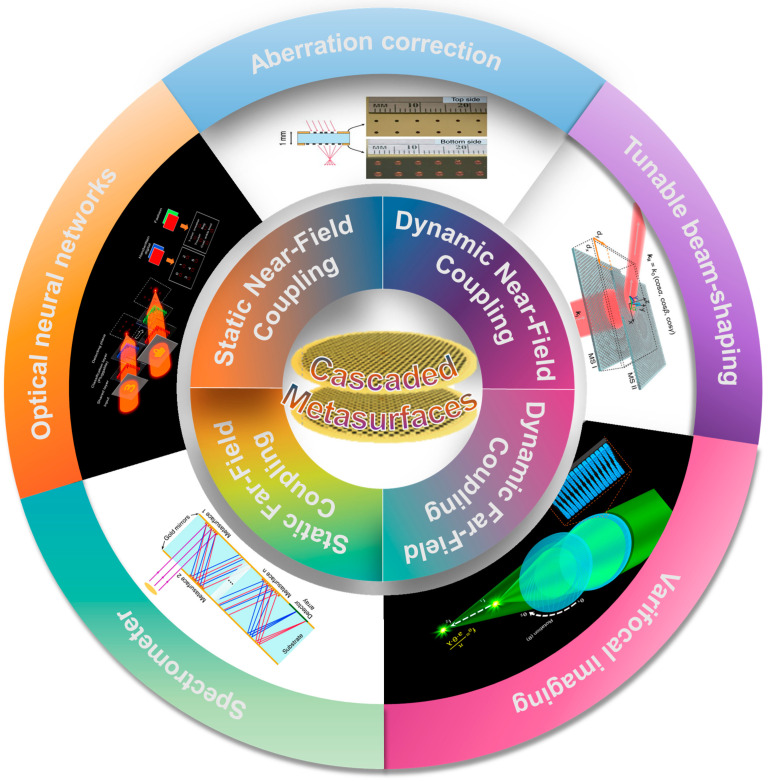
Categories and applications of cascaded metasurfaces. Reproduced with permission. Copyright 2016, published by Springer Nature [63]. Reproduced with permission. Copyright 2023, published by John Wiley and Sons [64]. Reproduced with permission. Copyright 2021, published by American Chemical Society [65]. Reproduced with permission. Copyright 2024, published by the Institute of Optics and Electronics [55]. Reproduced with permission. Copyright 2018, published by Springer Nature [66].

The past decade has witnessed significant advancements in single-layer metasurfaces with extensive exploration of various material systems, unit cell designs, and implementation strategies [41,42]. Furthermore, multifarious mechanisms for achieving tunable metaedevices were investigated including the utilization of phase-changing materials [43,44,45], liquid crystals [46], stretchable substrates [47,48,49,50], and micro-electro-mechanical systems (MEMS) [51,52]. It is a natural idea to extend single-layer metasurface to cascaded metasurfaces. Similar to traditional optical systems, cascading multiple layers of metasurfaces unlocks additional degrees of freedom and accomplishes complex tasks that are beyond the capabilities of single-layer metasurfaces alone, such as aberration correction [53], high-purity vortex beam generation [54], and optical neural networks [55] (Figure 1). It is worth noting that recent studies have demonstrated the efficacy of deep learning in predicting and designing metasurface functionalities, such as in the design of materials and structures for core–shell nanoparticles [56] and probabilistic representation and inverse design of metamaterials [57]. Additionally, data-driven approaches are increasingly utilized to explore vast parameter spaces, achieving design efficiency and robustness, as highlighted in recent advancements [58,59]. In recent years, cascaded metasurfaces and metadevices have garnered much attention, but they have rarely been summarized or reviewed; most of the related reviews just briefly mentioned cascade metasurfaces in general [60,61,62].

Therefore, this paper focuses on cascaded metasurfaces formed by serially combining or stacking two or more layers of metasurfaces with unit cells comprising typical plasmonic or all-dielectric resonators. The coupling mechanisms underlying these cascaded metasurfaces determine the corresponding design methodologies and application scenarios. We provide a comprehensive overview of recent advancements in related areas, delving into the intricate coupling mechanisms of metasurfaces based on near-field and far-field interactions while systematically analyzing their applications in both static and dynamic configurations. It is anticipated that this review will facilitate a profound comprehension of cascaded metasurfaces, showcase their immense potential in constructing advanced integrated devices, and serve as a valuable reference for future research.

## 2. Cascaded Metasurfaces Based on Near-Field Coupling

The coupling relationship in cascaded metasurfaces is determined by the distances between layers, which correspond to different theoretical models and design approaches. Near-field coupling occurs when the interlayer distances are sufficiently smaller than the working wavelength, causing strong EM near-field interactions between the stacked unit cells and activating new resonant modes. In essence, near-field coupling can be considered as a means of constructing novel types of unit cells. Near-field-coupled cascaded metasurfaces can be further divided into two categories: static cascading and dynamic cascading. In static cascaded metasurfaces, there is no relative motion between the layers during operation, and micro-fabricated thin films with fixed thickness can be used to maintain the interlayer spacing. Based on this approach, a terahertz polarization converter and an anomalous refractor have been proposed, constructed from a three-layer metasurface structure with each layer separated by subwavelength-thick polyimide [67]. Additionally, a four-layer cascaded structure has been utilized to design a transmissive metasurface lens for near-infrared wavelengths [68]. On the other hand, movable cascaded metasurfaces incorporate relative motions such as translation, rotation, or tilting between the layers. For example, beam scanning [69] and moiré responses [70] have been achieved through the control of the rotation of metasurfaces. However, these methods require a transfer printing process for each rotation angle at small working wavelengths [70], which is not suitable for real-time operation. The following subsections discuss this topic in detail.

### 2.1. Static Configuration

**Figure 2:**
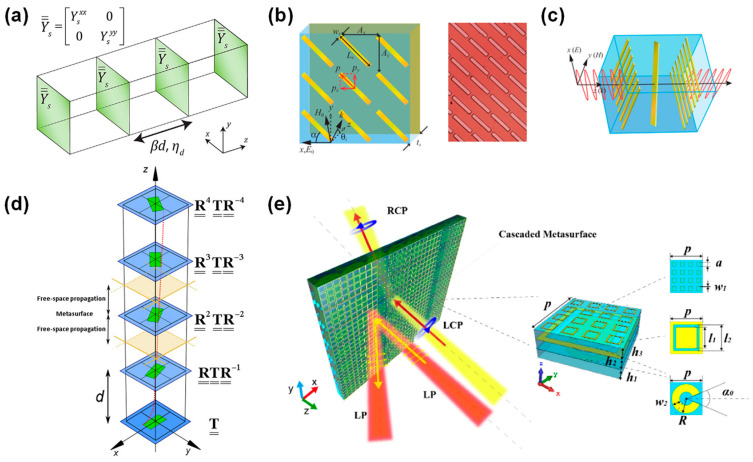
(**a**) A generic structure consisting of four cascaded metasurfaces (electric sheet admittances) separated by dielectric layers. In general, the sheet admittances are anisotropic such that *x*- and *y*-polarized light can be controlled independently [68]. (**b**) Broadband polarization conversion in reflection. Schematic (**left**) and microscopic image (**right**) of a metamaterial linear polarization converter [67]. (**c**) Broadband polarization conversion in transmission. Schematic of the unit cell of the metamaterial linear polarization converter, in which a normally incident *x*-polarized wave is converted into a *y*-polarized one [67]. (**d**) Schematic geometry of a twisted metamaterial. Each layer is rotated by a constant angle compared to its immediate neighbor. The transfer matrix of each twisted unit cell can be obtained by suitably rotating the transfer matrix of the first unit cell. A twisted unit cell consists of a propagation length *d* in free space and an ultrathin metasurface in the middle [72]. (**e**) Schematic model of the cascaded metasurface device and its meta-atom design. The meta-atom consists of three functional structure layers that are a reflection layer, a filtering layer, and two transmission layers [73]. (**a**) Reprinted/adapted with permission from Ref. [68]. Copyright 2013, American Institute of Physics (AIP). (**b**) Reprinted/adapted with permission from Ref. [67]. Copyright 2013, American Association for the Advancement of Science (AAAS). (**c**) Reprinted/adapted with permission from Ref. [67]. Copyright 2013, American Association for the Advancement of Science (AAAS). (**d**) Reprinted/adapted with permission from Ref. [72]. Copyright 2014, American Physical Society (APS). (**e**) Reprinted/adapted with permission from Refs. [67,73]. Copyright 2019, Optical Society of America (OSA).

Pfeiffer et al. accomplished complete control over the phase and polarization of light waves by cascading four metasurface layers with inductor–capacitor units (Figure 2a) [68]. Compared to traditional single-layer metasurface designs, this multilayer cascading approach significantly enhanced transmission efficiency in the near-infrared range (at a working wavelength of 2 μm), with a simulated peak intensity that was 62% of an ideal lens of the same dimensions, which is unavailable to single-layer plasmonic resonators due to severe ohmic loss and reflection (focusing efficiency typically no larger than 10%) [71]. Each metasurface layer, designed with anisotropic units, can independently control light waves of different polarization states, achieving extreme birefringence effects within a subwavelength thickness. This design can generate quarter-wave plates to convert linearly polarized light into circularly polarized light. The near-field coupling mechanism of the static cascaded metasurface is realized through the inductive and capacitive coupling of each layer, playing a key role in enhancing the phase control accuracy and transmission efficiency.

Grady et al. proposed a cascaded metasurface structure that realizes simultaneous linear polarization conversion and wavefront shaping, where the metal cut-wire array structure is utilized to achieve a 90° rotation of polarization direction (Figure 2b) and a gradient plasmonic nano-antennae array is employed to realize anomalous refraction in the terahertz band (Figure 2c) [67]. This structure, through the precise design of the metasurface, can efficiently operate over an extremely broad bandwidth, while between 0.8 and 1.36 THz, the cross-polarized reflection is higher than 80% and the co-polarized one is below 5%. The traditional design demonstrates a bandwidth of ~0.1 THz [74]. The metal cut-wire structure controls the phase and polarization of EM waves by generating electric dipole resonances. This efficient conversion is due to the multiple reflections and near-field coupling effects within the Fabry–Pérot cavity structure. Specifically, the incident wave excites electric dipoles in the cut wires, and these dipoles’ currents produce resonance modes that switch between different resonant modes through near-field coupling. Experiments showed that in the frequency range of 0.7 to 1.9 THz, the cross-polarized reflection can exceed 80%, realizing broadband and efficient linear polarization conversion.

Askarpour et al. developed a generalized Floquet analysis method to study wave propagation in twisted metamaterials, which are composed of a series of identical planar metasurfaces with sequential rotations(Figure 2d) [72]. Their analysis revealed how the modal dispersion in these metamaterials inherently differs from that of conventional periodic structures and how the eigenmodes support specific circular polarization properties based on lattice effects. Their study discussed how static twisted metamaterials can regulate EM waves through near-field coupling. The researchers demonstrated that by superimposing multiple twisted structures, precise control over the phase and amplitude of the incident waves could be achieved. This design is significant for beam manipulation in the terahertz band.

Yang et al. further explored how cascaded metasurfaces can simultaneously control the transmission and reflection of EM waves in a static state [73]. They proposed a cascaded metasurface that integrates resonant phase unit cells and geometric phase unit cells, capable of independently manipulating transmitted and reflected waves at different frequency bands (Figure 2e). By designing specific phase distributions, the metasurface can reconstruct the wavefront of reflected and transmitted waves within shared apertures across different frequency bands. The reflection beam can be effectively manipulated within the 10.8–11.6 GHz frequency band, providing a bandwidth of 0.8 GHz. Meanwhile, the transmission beam is precisely controlled in the 6.1–6.3 GHz band, with a bandwidth of 0.2 GHz. The near-field coupling between multilayer metasurfaces enhances the phase modulation capability of the EM waves within the structure. Each metasurface layer, designed with different resonators, generates distinct resonant responses to the incident wave, and the near-field coupling between layers further adjusts the phase distribution of transmission and reflection waves. This approach allows for flexible control over transmission and reflection characteristics without altering the overall material structure.

In summary, static cascaded metasurfaces, formed by stacking multiple metasurface structures with near-field coupling effects, can efficiently control the phase, polarization, and transmission/reflection characteristics of EM waves. The interactions between layers give rise to resonant modes that are unavailable to single-layer structures, not only enhancing the overall system’s transmission efficiency but also enabling a variety of EM multifunctionalities.

### 2.2. Dynamic Configuration

**Figure 3:**
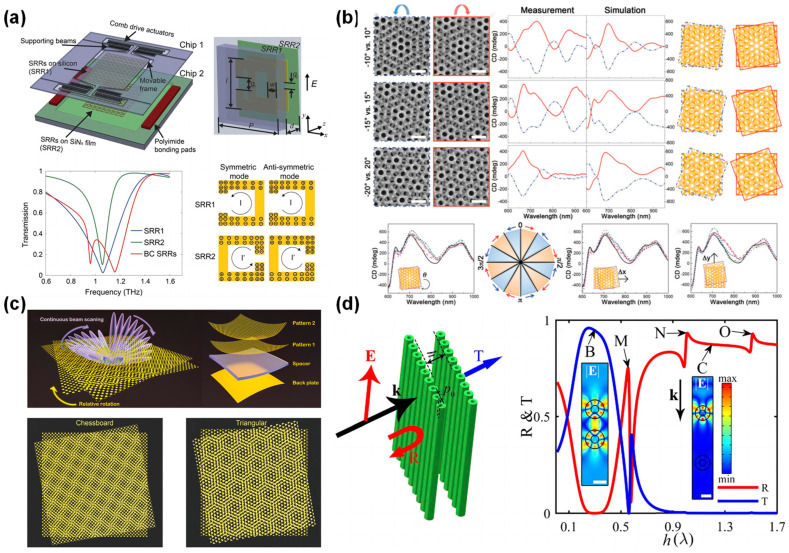
(**a**) Exploded view of tunable metamaterials (**upper left**). One unit cell of the tunable metamaterial, including BC-SRRs, where SRR1 is on a silicon frame and SRR2 is on a SiNx thin film (**upper right**). Simulated spectra of the individual uncoupled SRRs and broadside-coupled SRRs when *d* = 20 μm. (**lower left**) The surface charge distribution of the symmetric mode and antisymmetric mode of BC-SRRs (**lower right**) [75]. (**b**) Tunable chiroptical properties of MCMs. SEM images (**Upper left**), measured and simulated CD spectra (**upper middle**), and schematic illustrations of three sets (i.e., −10° vs. 10°, −15° vs. 15°, and −20° vs. 20°, respectively) of MCMs (**upper right**). The scale bars are 1 µm. A series of CD spectra of an MCM under various rotation angles from 15° to 315° at an interval of 60°, a wheel illustration of the rotational periodicity (π/3) in the θ-dependent chiroptical properties of the MCMs, and a series of CD spectra of an MCM under the various lateral translations of the top layer of Au nanohole arrays in the *x* and *y* directions, respectively, from 0 to 500 nm with an interval of 100 nm (**Lower left to right**) [70]. (**c**) Moiré metasurface in real space. Schematic illustration of a moiré metasurface (**upper**). The mutual twist of two closely attached metasurfaces produces a varying moiré pattern. Composition of a moiré metasurface from bottom to top: a metallic back plate, a spacer, and two closely stacked metasurface layers with a mutual twist (**lower**) [69]. (**d**) Schematic of a bilayer metasurface stacked by two MMs with interlayer spacing *h* (**left**). *k* is the incident wavevector. Reflection (R) and transmission (T) spectra of the bilayer metasurface as a function of *h* (**right**). The insets correspond to the |***E***| distributions of points B and C, respectively [76]. (**a**) Reprinted/adapted with permission from Ref. [75]. Copyright 2016, Nature Publishing Group (NPG). (**b**) Reprinted/adapted with permission from Ref. [70]. Copyright 2017, Wiley-VCH. (**c**) Reprinted/adapted with permission from Ref [69]. Copyright 2022, American Association for the Advancement of Science (AAAS). (**d**) Reprinted/adapted with permission from Refs. [67,76]. Copyright 2022, Optical Society of America (OSA).

Zhao et al. proposed a voltage-controlled, dynamically tunable, double-layer terahertz metamaterial based on MEMS and broadside-coupled split-ring resonators (BC-SRRs) [75]. (Figure 3a) This system utilizes a comb-drive actuator to provide up to 20 μm of lateral displacement, modulating the continuous interaction between the coupled resonators. The study demonstrated that near-field coupling in split-ring resonators results in mode splitting between symmetric and antisymmetric modes. As the relative position changes, the frequency of the modes shifts significantly. The symmetric mode’s frequency shifts to higher frequencies with increasing lateral displacement, while the antisymmetric mode shifts to lower frequencies. This frequency tuning allows for precise control of the amplitude and phase of terahertz waves, achieving 74% amplitude modulation at 1.03 THz and a 180° phase shift at 1.08 THz.

Wu et al. reported a new type of chiral metamaterial—moiré chiral metamaterials (MCMs)—which consists of two identical achiral gold nanohole arrays stacked into moiré patterns [70] (Figure 3b). The optical chirality of these metamaterials originates from the relative in-plane rotation between the two layers. By precisely controlling the in-plane rotation angle between the two layers of nanohole arrays, the chiral response can be tuned, enabling label-free enantio-discrimination of biomolecules and drug molecules at the picogram level. This showcases ultrathin (about 70 nm), strong chirality, and highly tunable moiré chiral metamaterials.

Liu et al. introduced a metasurface for dynamic beamforming based on the moiré effect [69] (Figure 3c). This metasurface utilizes the mutual rotation of two closely stacked metasurface units to form a moiré pattern with low spatial frequency, achieving dynamic control of the reflected beam. This geometric pattern-based dynamic control relies on near-field coupling between layers and the structure’s response to EM waves. The moiré pattern formed by two periodic patterns varies with different rotational angles, and as the patterns rotate relative to each other, the direction and intensity of the beam change. The near-field coupling of this structure affects its interaction with the incident wave, enabling various beam control functions. By adjusting the relative position of the patterns at different angles, researchers successfully demonstrated continuous beam steering from 8.7° to nearly 90°.

Song et al. proposed a tunable bilayer metasurface composed of two stacked identical dielectric magnetic mirrors (Figure 3d) [76]. The structure relies on strong near-field EM coupling between the two layers, which can be precisely controlled by adjusting the spacing and relative position between the layers to achieve frequency tuning and phase control. Under specific conditions, such as when the layer spacing *h* = 0.302*λ*, an almost perfect conversion occurs. The tunable bilayer periodic metasurface exhibits a transmission rate adjustable range from approximately 0.01 to 0.74. In the high transmission mode, the operating bandwidth is superior to that of single-layer structures. The reversible conversion between high reflection and high transmission was demonstrated, showing that the high transmission characteristic of the bilayer metasurface is robust against positional perturbations.

In summary, dynamic cascaded metasurface devices based on near-field coupling provide dynamic control over the propagation characteristics of EM waves through the precise design of inter-unit interactions. Due to the required deep-subwavelength interlayer spacing, this method is more suitable for larger wavelength bands. Future work could involve stacking more layers of metasurfaces to achieve more complex beam control and dynamic tuning functions. Additionally, the use of machine learning technology to optimize structural design also offers more possibilities for the study of dynamic tuning through near-field coupling.

## 3. Cascaded Metasurfaces Based on Far-Field Coupling

Far-field-coupled cascaded metasurfaces exhibit significant differences in design methodology compared to near-field coupling. When the interlayer spacing of metasurfaces is sufficiently large, the near-field effects between adjacent layers can be neglected, allowing the use of traditional optical design methods such as ray tracing and diffraction integral. In essence, far-field-coupled cascaded metasurfaces can be regarded as stackings of traditional single-layer metasurfaces with classic plasmonic or dielectric unit cells. In this scenario, the entire system should be considered in advance, optimizing the phase profile and interlayer spacing for each metasurface layer for targeted functionality. Then, each individual metasurface can be designed with theories and methods similar to those used for single-layer metasurface design [63].

Similar to situations of near-field coupling, far-field-coupled cascaded metasurfaces can also be categorized into static cascaded and dynamic cascaded types. For static cascaded metasurfaces, multiple metasurface layers can be fabricated on both sides of a substrate of a certain thickness [77] or fixed using mechanical fixtures [78]. For example, diffraction-limited focusing and imaging of monochromatic light within a ±25° incident angle range in the visible light spectrum has been achieved by iteratively optimizing the phase distribution of metasurfaces through ray tracing [79]. In another study, cascaded metasurface retroreflectors were designed via iterative ray tracing optimization, enabling incident light from a particular angle to be completely reflected to its original path [80]. Compared to near-field coupling, far-field coupling allows metasurfaces to have interlayer spacings on the order of thousands of times of the working wavelength, thereby reducing the precision requirements for controlling the spacing and simplifying the integration with actuators to achieve dynamically tunable systems. For instance, some researchers have achieved varifocal lenses [81,82,83] and beam scanning functions [64] by controlling and adjusting the translation of cascaded metasurfaces. Despite the many advantages of far-field-coupled cascaded metasurfaces, there are also limitations: the large interlayer spacing weakens the compactness brought by the ultrathin characteristics of metasurface to some extent [84]. Therefore, to optimize the design of far-field-coupled metasurfaces, it is still necessary to seek better solutions that balance interlayer spacing with functional performance. The following subsections discuss this topic in detail.

### 3.1. Static Configuration

**Figure 4:**
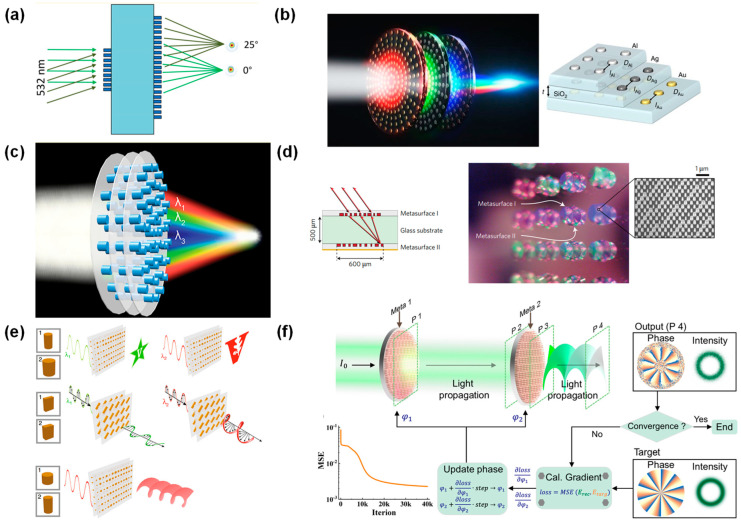
(**a**) Schematic diagram of the metalens doublet [79]. (**b**) Three-layer lens. (**left**) Artist’s view of the three-layer lens. When illuminated with white light, each layer focuses its designated part of the spectrum to a distance of 1 mm along the optical axis. (**right**) Schematic illustration of the layered structure. Each layer consists of nanodiscs with the following diameters D and separations l: DAu 1⁄4 125 nm, lAu 1⁄4 185 nm; DAg 1⁄4 85 nm, lAg 1⁄4 195 nm; DAl 1⁄4 120 nm, lAl 1⁄4 150 nm [86]. (**c**) The multiwavelength metalens doublet (NA = 0.42) [84]. (**d**) A monolithic planar retroreflector made of two metasurfaces. Schematic drawing of the planar retroreflector (**left**). Two metasurfaces are patterned on opposite sides of a glass substrate. Optical image of an array of retroreflectors (**right**) [80]. (**e**) Illustrations of multiwavelength holograms (**upper**), multiwavelength waveplates (**central**), and 3D holograms (**lower**) using bilayer metasurfaces [87]. (**f**) Working principle and inverse design realized with cascaded metasurfaces. A schematic of cascaded metasurfaces composed of numerous TiO2 nanorods (**upper**). Starting with a random phase, the optimization loop eventually converses with the target fields with a very small MSE (**lower**) [54]. (**a**) Reproduced with permission from Ref. [79]. Copyright 2017, Nano Lett. (**b**) Reproduced with permission from Ref. [86]. Copyright 2017, Nat. Commun. (**c**) Reproduced with permission from Ref. [84]. Copyright 2018, Nano Lett. (**d**) Reproduced with permission from Ref. [80]. Copyright 2017, Nat. Photonics. (**e**) Reproduced with permission from Ref. [87]. Copyright 2019, Light: Sci. Appl. (**f**) Reproduced with permission from Ref. [54]. Copyright 2023, Nat. Commun.

One of the most important capabilities of the far-field-coupled static cascaded metasurface is aberration and distortion correction. As early as 2016, Arbabi et al. developed a miniature wide-angle camera incorporating metasurface doublets [63]. The two cascaded metasurfaces are specifically designed to correct monochromatic aberrations for wide-angle imaging, enhancing the overall optical performance. Their far-field coupling enables an independent yet complementary function, effectively minimizing monochromatic aberrations across a broad angle. This innovation paves the way for compact imaging systems, particularly suited for mobile cameras and portable devices. To correct aberrations such as spherical and coma aberrations for compact camera systems, the visible-wavelength metalens doublet shown in Figure 4a was reported [79]. This metalens doublet has a numerical aperture of 0.44, a focal length of 342.5 μm, and a field of view of 50° that enables diffraction-limited monochromatic imaging along the focal plane at a wavelength of 532 nm. Recently, Zheng et al. extended the cascaded metasurface imaging concept to achieve distortion-free imaging [85]. Their compound metalens utilizes far-field coupling among multiple metasurfaces, each tailored to correct specific aberrations. This setup not only eliminates distortion but also maintains a wide field of view and diffraction-limited performance. By stacking these metasurfaces, they achieved superior image quality over single-layer designs, demonstrating the potential of cascaded metasurfaces for imaging systems demanding minimal optical distortion. Fu et al. proposed step-zoom metalenses for optical systems that can have two focal lengths depending on the state of polarization, enabling step-zoom functionality without optical and mechanical compensation [77].

The unit cells of metasurfaces enable subwavelength optical wave manipulation but often suffer from severe phase dispersion. To address this, researchers have proposed stacking independent metasurface layers, each tailored for a distinct spectral band. A notable example is the achromatic metalens for red, green, and blue, demonstrating functional beam shaping and anomalous dispersive focusing. Avayu et al. introduced layered metasurfaces optimized for multispectral achromatism, effectively multiplexing spectra while minimizing crosstalk [86]. Their RGB triplet lens represents a great progression in static metasurface configurations, suitable for microscopy, imaging, and displays. By leveraging far-field interactions, the stacked metasurfaces reduce chromatic dispersion and enhance multiwavelength focusing, ushering in ultrathin, super-achromatic optical elements with diverse functionalities. The three-layer metasurface lens is shown in Figure 4b.

Chromatic aberration can also be corrected by the synthetic design of cascaded metasurfaces. Feng et al. presented a red–green–blue (RGB) achromatic metalens doublet with a 1 mm diameter and a high numerical aperture (NA) of 0.8 [88]. Designed to correct chromatic aberrations at discrete RGB wavelengths, the metalens achieves high focusing efficiency with a simplified bilayer structure, improving the ease of fabrication. The doublet employs two distinct meta-atom shapes, ensuring RGB wavelengths converge at a single focal plane and enhancing digital imaging applications such as projectors, VR, and microscopy. Testing confirmed its capability for high-quality color imaging, with potential applications in advanced imaging systems. In 2018, Zhou et al. proposed the use of multilayer dielectric metasurfaces to improve the efficiency of multiwavelength operation [84]. As a proof of concept, the study demonstrated a multiwavelength metalens triplet (NA = 0.42) with focusing efficiencies of 38% and 52% at wavelengths of 1180 and 1680 nm, respectively, as shown in Figure 4c. Tang et al. proposed a metasurface doublet design that corrects chromatic aberration and focuses light at wide incident angles [89].

In addition to aberration correction, static cascaded metasurfaces based on far-field coupling demonstrate a wide variety of novel optical functions. A notable example comes from Arbabi et al., who developed a planar metasurface retroreflector in 2017 [80], as shown in Figure 4d. Unlike a single metasurface, the paper suggests the use of two stacked metasurfaces, one of which performs a spatial Fourier transform and the other imparts a spatially varying momentum to the light, resulting in a wide field of view and efficient retroreflection. The retroreflector has a normal incidence efficiency of 78% and a half-power field of view of 60°, which is larger than conventional designs. In terms of applications, the lightweight and planar nature of this retroreflector makes it suitable for optical free-space communications, laser tracking, dynamic optical labeling, and remote sensing.

The development of a compact metasurface spectrometer and its advantages in terms of reduced size and high resolution are attracting researchers’ attention. Faraji Dana et al. introduced the concept of folded metasurface optics, showcasing a compact spectrometer realized within a 1 mm thick glass slab (7 mm³) that leverages reflective cascaded metasurface [66]. The spectrometer features a resolution of ~1.2 nm, resolving over 80 spectral points within 760–860 nm, achieved through three reflective dielectric metasurfaces monolithically fabricated in a single lithography step. This design, where light is confined and wavefronts controlled within reflective interfaces, presents a promising avenue for downsizing and enhancing the robustness, integration potential, and versatility of optical systems like signal processors, interferometers, hyperspectral imagers, and computational optics.

Cascaded metasurfaces are also employed to enhance beamforming capabilities. An all-dielectric metasurface double beam was presented by Zhou et al., which enables precise beam manipulation for telecommunication systems [90]. The development of a planar telescope capable of polarization-controlled enhanced beam steering provided a significant advance in beam manipulation for LiDAR and optical systems [91]. As early as 2014, there were studies on the generation of cylindrical vector vortex beams by two cascaded metasurfaces [92]. While in 2023, Mei et al. demonstrated a new approach using an optical neural network and cascaded pure-phase element surfaces, as shown in Figure 4f, to generate ultrapure Laguerre–Gaussian vortex beams (max (l = 200)) in a static setup with a radial purity of 96.71% and an efficiency of 70.48% [54]. Their system, comprising two metasurfaces optimized via optical neural networks, suppresses backward reflection and efficiently converts Gaussian beams to high-order vortex beams. This breakthrough, leveraging Rayleigh–Sommerfeld diffraction for far-field coupling, enhances optical device functionality and significantly impacts optical communication and quantum optics, where vortex beam quality is paramount. The cascaded design, correcting intensity and phase distortions sequentially, represents a major advancement over single-layer metasurface technologies.

Cascading metasurfaces with polarization or wavelength-tailored responses provide optical manipulation multifunctionality within the static configuration. Dual-layer dielectric metasurfaces emerge as a promising platform to overcome limitations in single-layer designs, enabling independent manipulation of the phase, amplitude, and polarization of light. By allowing independent geometry and function customization of each layer, multifunctional meta-optics can be realized, where two or more optical properties can be tailored independently. Demonstrations of multiwavelength holograms, multiwavelength waveplates, and 3D holograms showcase this potential, as shown in Figure 4e [87]. Yu et al. reported a spin-controlled metasurface doublet that functions as a camera, microscope, and telescope by varying the polarization of incident light [93]. Bao et al. demonstrated the successful realization of a spatially varying Jones matrix with the full eight degrees of freedom (DOFs) in linear optics [94]. By cascading two metasurface layers and leveraging gradient descent optimization, the authors achieved this multifunctionality numerically and experimentally in optical frequencies. This ultimate control over the Jones matrix parameters unlocks new avenues for designing optical functionalities that were previously unattainable, promising broad potential applications across various optical domains. Research by Gao et al. on the dual-layer aspect of dielectric polarization-filtering metasurfaces can be used for triple-function control of light, including beam deflection, focusing, and vortex generation [95].

Cascaded metasurfaces may also accelerate the development of optical computing. An innovative all-optical controlled-NOT (CNOT) logic gate reported by Huang et al. utilizes a metasurface doublet that enables directional asymmetric EM transmission [96]. By manipulating circularly polarized light based on its spin and direction, this design overcomes the limitations of traditional optical gates, offering compactness and robustness. The system, verified experimentally, demonstrates multiple input–output states corresponding to CNOT operations in the infrared, with potential applications in optical computing, chiral imaging, and beyond. Its geometric optics-based approach renders it adaptable to different spectra, hinting at a versatile platform for advancing EM control in various fields.

In summary, cascaded metasurfaces are transforming optics by enabling multifunctional, compact, and precise optical systems through far-field coupling and computational design. These systems overcome traditional limitations, allowing for highly efficient chromatic and monochromatic aberration correction, wide-angle and distortion-free imaging, and versatile beam shaping. Novel applications span from lightweight retroreflectors for optical communications to compact spectrometers and beamforming devices for LiDAR and telecommunication systems. Recent breakthroughs, such as the development of an all-optical CNOT gate, highlight potential advancements in computational optics. The continuous evolution of cascaded metasurfaces demonstrates their expanding role in achieving unprecedented optical functionalities across diverse fields.

### 3.2. Dynamic Configuration

Dynamic far-field coupling represents a prevailing tunning mechanism of tunable metasurfaces. The design of dynamic far-field coupling involves rotation and translation to achieve precise control over optical properties like focal length and beam direction.

Research on rotational cascaded metasurfaces has made significant progress in recent years, which leverages the relative rotation of stacked metasurface layers to change the overall phase profile. Rotational adjustments can enhance a system’s ability to manipulate wavefronts [97], making them ideal for applications in adaptive optics and multi-foci imaging [98]. This design principle has been widely used in different bands, including visible light [99], infrared light [100], and terahertz bands [26,65,101,102], demonstrating its wide spectral applicability.

**Figure 5:**
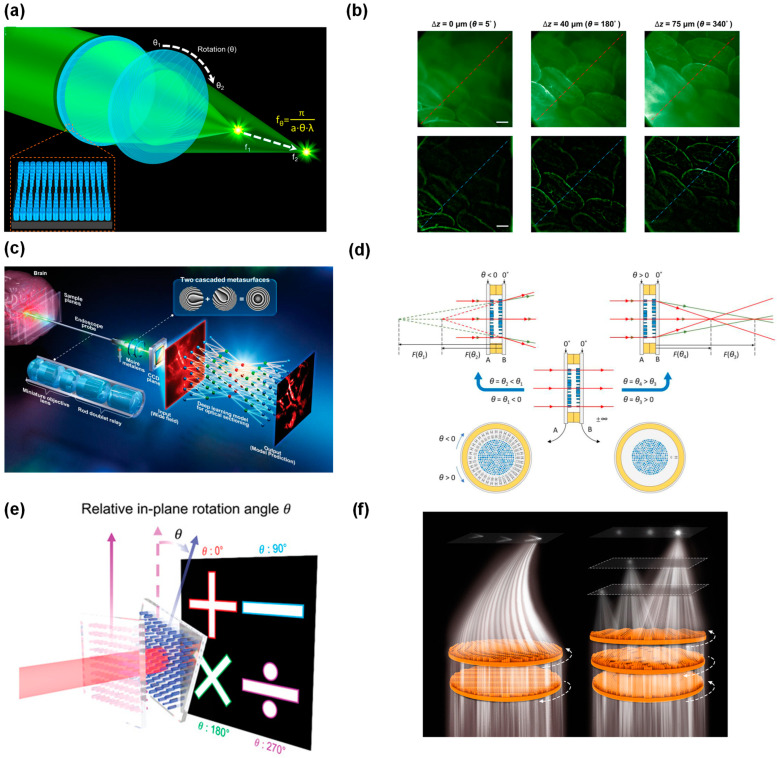
(**a**) Schematic of moiré metasurface operation [65]. Reproduced with permission. Copyright 2021, American Chemical Society. (**b**) Three uniformly illuminated facial fluorescence images at different depths and processed with HiLo [65]. (**c**) Schematic of the varifocal metalens based intelligent fluorescence endo-microscopy [105]. Reproduced with permission. Copyright 2024, John Wiley and Sons. (**d**) Scheme of the zooming imaging doublet consisting of two metasurfaces. The focal length of the doublet changes continuously when varying the relative angle θ. For θ > 0, the doublet works as a positive lens, and for θ < 0, as a negative lens [106]. Reproduced with permission. Copyright 2024, John Wiley and Sons. (**e**) Rotational multiplexing method for cascaded metasurface holography, adapted from Wei et al. [26]. Reproduced with permission. Copyright 2024, John Wiley and Sons. (**f**) By rotating multiple metasurfaces, the focal spot can be dynamically controlled in both two-dimensional (2D) and three-dimensional (3D) spaces [102]. Reproduced with permission. Copyright 2023, The American Association for the Advancement of Science.

The moiré lens is a typical varifocal design based on relative rotation between bilayer elements, which has attracted attention in traditional DOE (diffractive optical element) research [103,104]. Below is an illustration of the basic principle of moiré metasurface operation, showing how the focal length changes with relative rotational angles (Figure 5a). In the field of biological imaging, zoom moiré lenses are used to realize optical sectioning fluorescence microscopy. By fine-tuning the focal length through rotation, these lenses, designed for optical sectioning in fluorescence microscopy by Luo et al., provide precise control over imaging depth and focus displacement, making them ideal for real-time dynamic imaging [65] (Figure 5b). For visible wavelengths, Ogawa et al. designed a rotational varifocal moiré metalens made of octagonal single-crystal silicon pillars [99]. Recently, Chia et al. introduced a deep learning assisted fluorescence endo-microscopy system designed for in vivo 3D imaging of internal tissues, particularly the brain. The system achieves a large field of view, invariant magnification, and deep tissue penetration, paving the way for faster, more accurate in vivo imaging in medical and research applications [105]. A schematic of the varifocal metalens is shown in Figure 5c.

Recent research has introduced advanced design methods that significantly improve the performance of rotational cascaded metasurfaces. For instance, the enhanced moiré metalens design proposed by Wei et al. has phase compensation and is able to vary the focal length by adjusting the relative rotational angle between two metasurfaces. The use of silicon nanocylinders on silica substrates ensures that the lens works independently of the polarization of incoming light; Figure 5d illustrates this concept [106]. To further address efficiency loss due to phase distortion when the spacing between two metasurfaces is large, a reinforced design reported by Qian et al. rectifies phase distortions and maintains a high focusing efficiency, 3.4 times higher than traditional designs [107]. Additionally, rotational cascaded metasurfaces have enabled dynamic holography and optical encryption, significantly expanding their utility in data security and storage [108]. For instance, a rotational multiplexing method based on cascaded metasurface holography is introduced in Figure 5e, which allows the generation of multiple independent holographic images by varying the relative rotation of stacked metasurfaces [26]. Another breakthrough comes from the work of Wang et al., who introduced a moiré metadevice that enables the dynamic generation of high-order Bessel beams (HOBBs) via the rotation of cascaded metasurfaces [109]. This unique capability provides potential applications in optical quantum communications, particle manipulation, and high-resolution imaging, where stable, long-range optical beams are necessary. In addition, metasurface lenses in the terahertz band are also used in far-field beam control and wavefront shaping technology [101], unlike conventional THz devices that rely on active elements for local tuning the cascaded metasurfaces, where wavefront control is achieved by rotating metasurfaces at different speeds, dynamically adjusting the effective Jones matrix of the device. With the development of communication technology, in order to solve the transmission direction and signal strength control problems of 6G communication technology, Zhang et al. proposed a cost-effective 3D varifocal metadevice. The metadevice utilizes rotational doublet (Airy beams) and triplet (Gaussian beams) to enable flexible and continuous control of THz waves in 3D space (shown in Figure 5f), ensuring that signal transmission is highly focused and directionally tunable [102].

In-plane translation refers to the lateral movement of metasurface layers, which significantly shifts the overall optical properties of a cascaded metasurface device [110]. When a metasurface undergoes in-plane translation, the unit cells move parallel to the metasurface plane, altering the phase profiles. This movement can modulate light–matter interaction areas, enabling tunable beam deflection or focusing without changing the axial position.

**Figure 6:**
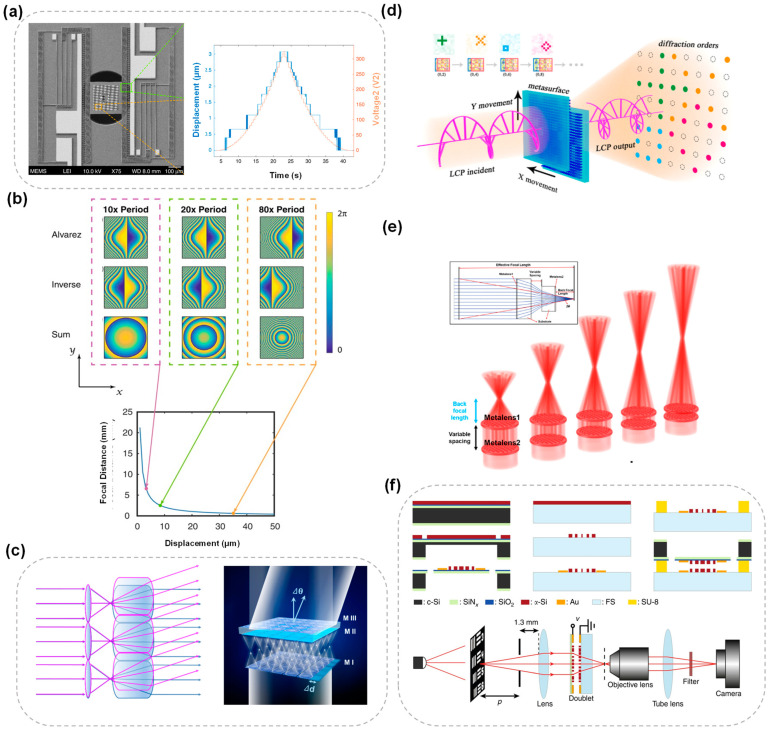
(**a**) SEM and optical images of a MEMS-actuated Alvarez metalens. The right side shows the actuated focal displacement and applied voltage over time. The general trend of the actuated displacement follows the square of the applied voltage [112]. Reproduced with permission. Copyright 2020, Springer Nature. (**b**) Behavior of the Alvarez lens in response to x displacement, adapted from Zhan et al. [113]. Plot of focal length dependence on displacement. Larger displacements result in a more rapidly varying phase profile, corresponding to a lens with a smaller focal length [113]. Reproduced with permission. Copyright 2017, Springer Nature. (**c**) Schematics of the decentered microlens array-based BSS [119]. Reproduced with permission. Copyright 2022, American Chemical Society. (**d**) Illustration of diffraction pattern switch by using cascaded metasurfaces, by moving one piece in the *x* or *y* direction, the corresponding diffraction orders can be switched [120]. Reproduced with permission. Copyright 2024, John Wiley and Sons. (**e**) Schematic of the zoom metalens. Inset: detailed layout of the zoom metalens [121]. Reproduced with permission. Copyright 2024, American Chemical Society. (**f**) Fabrication process summary and schematic illustration of the imaging setup using a regular glass lens and the tunable doublet. The image formed by the doublet is magnified and re-imaged using a custom-built microscope with a ×55 magnification onto an image sensor. A simplified fabrication process of a lens on a membrane (**left**), a simplified fabrication process of the lens on the glass substrate (**middle**), and schematics of the bonding process (**right**) [83]. Reproduced with permission. Copyright 2018, American Chemical Society.

A key example of this approach is the varifocal Alvarez lens, allowing for rapid change in the focal length achievable with small physical displacement [111]. For example, a MEMS-actuated 6.3 μm lateral shift can result in a focal length change of 68 μm, as shown by Han et al. [112], who explored a tunable optical lens system that uses metasurface optics and MEMS actuation for focal length modulation, providing a fast and precise method through comb-drive structures to tune the focal length without mechanical movement along the optical axis. The specific micro–nano-structure SEM image is shown in Figure 6a. Additionally, these systems offer a higher degree of miniaturization compared to conventional optics, as the lateral movement allows for significant optical power adjustments without altering the physical thickness of the device. Moreover, in-plane translation metasurface lenses have also shown remarkable capabilities in extending the depth of focus. In some designs, such as the Alvarez metasurfaces system reported by Zhan [113] et al., a ~300 μm deep field is achieved by combining the system with a cubic phase plate while minimizing chromatic aberrations, which is critical for broad-spectrum applications, including white-light imaging. The behavior of the Alvarez lens in response to x displacement is shown in Figure 6b. The metasurface Alvarez lens reported offers a tunable focal length over a range of 2.5 mm with only 100 μm of mechanical displacement, highlighting potential applications in ultra-miniature optical systems and advanced display technologies, such as augmented and virtual reality. In addition to using the Alvarez lens principle, Wang et al. presented a design for a continuous-zoom tunable bifocal metalens capable of controlling two foci with different focal lengths using cascaded bilayer metasurfaces. The relative intensity of the foci can also be adjusted by modulating the ellipticity of the incident light [114].

Not only in Visible Wavelength Operation but also in the infrared spectrum, a study by Colburn et al. demonstrated a high-throughput metasurface lens system with a 1 cm aperture and a focal length range exceeding 6 cm at 1550 nm [82]. This was achieved through lateral displacement of the metasurfaces by a few millimeters, highlighting the significant modulation capability of such designs. The lens also demonstrated a focusing efficiency of 57%, showcasing the potential for high-performance optical systems. There are also metasurface lens systems working in the microwave frequency band (C-band, centered at 7.5 GHz) [115]. Their application is tailored for lower-frequency EM waves, such as in microwave imaging, radar, and telecommunications. Hada et al. focused on the development of a varifocal metalens with polarization separation functionality, enabling the development of compact and highly functional optical devices suitable for advanced communication technologies [116]. Colburn et al. integrated metasurfaces with wavefront coding techniques, enabling high-quality imaging with adjustable focal lengths across different wavelengths [117]. They introduced two quartic phase metasurfaces that create a tunable extended depth-of-field lens capable of spectrally invariant focusing across the visible spectrum. A post-capture deconvolution algorithm is employed to enhance imaging quality, allowing for a full-color zoom imaging range of 5×. In order to solve the chromatic aberration problem in the wide wavelength range, Che et al. introduced a broadband achromatic Alvarez metalens, which remains free of chromatic aberration throughout the zooming process [81]. Unlike traditional design methods that are usually based on specific functions (such as quadratic and cubic phase distributions), the Integral of Total Phase Profile Difference (ITPD) method reported by Che et al. [118] provides a more general phase design framework that can be applied to a variety of optical systems, especially in situations where complex phase control and multi-functional integration are required.

In-plane translation of cascaded metasurfaces also gives rise to efficient beam steering capabilities. Our group introduced a novel design paradigm for tunable cascaded metasurfaces capable of continuous two-dimensional beam steering, featuring wide-angle tunability and minimal beam divergence. By leveraging a ray-tracing-based approach, this method enables effective operation with larger interlayer gaps, thus facilitating practical applications in fields such as LiDAR and free-space optical communication [64]. Chen et al. integrated micro-metalenses into a beam steering setup, offering high precision and control over the beam’s direction [119], a large field of view, and faster beam steering compared to conventional beam controllers. Schematics of the decentered microlens array (MMLA)-based beam steering are shown in Figure 6c, consisting of a single layer of MMLA (M I) and a cascading MMLA doublet (M II and M III). The gap between M I and M II is 158 μm. MI, M II, and M III share the same size at 177 μm × 177 μm. All of the metalenses are composed of silicon nano-posts.

In terms of controlling the diffraction pattern, Zhang et al. optimized the energy distribution in the diffraction pattern by combining Dammann optimization with a gradient descent algorithm based on TensorFlow and generated several distinct diffraction patterns, which are controlled by precise pixel-level alignment between the cascaded layers [120]. An illustration of a diffraction pattern switch by using cascaded metasurfaces is shown in Figure 6d. The switchable diffraction patterns can be used for precise optical scanning systems, offering high accuracy and flexibility in different scanning conditions.

Out-of-plane translation in cascaded metasurfaces refers to the movement of the layers along the axis perpendicular to the layer plane, typically altering the device’s focal length. Zhang et al. [121] developed a zoom metalens system that achieves a focal length range of 1.4 mm to 7.5 mm by adjusting the axial spacing between lenses by just 0.109 mm (Figure 6e). This system utilizes a polynomial phase profile to minimize optical aberrations and significantly extends the depth of focus, achieving a zoom range of up to 11.9×. In 2018, an innovative approach to tunable optics through the integration of metasurfaces and MEMS was reported by Arbabi et al. [83]. This design features a metasurface doublet with converging and diverging lenses that can be electrically tuned, allowing precise control over the focal distance and fast response times, as shown in Figure 6f. Such advancements are crucial for compact imaging systems in drones, microrobots, and other performance-sensitive applications.

## 4. Discussion

In the history of science and technology, numerous devices and systems have undergone a progression from simplicity to complexity and from sole operation to collaborative functionality, and metasurfaces are no exception. As a novel generation of optical elements, metasurfaces offer unprecedented design flexibility within a slender form factor, and their cascading makes fascinating possibilities for future research. In this review, we classify cascaded metasurfaces based on the presence of interlayer motion, and dynamic coupling is regarded as a tunning mechanism of tunable metasurfaces. Various other tunning mechanisms can also be incorporated into the cascaded metasurface configuration to unlock additional design routes. We trust that cascaded metasurfaces will contribute significantly to a wide range of optical applications and change the way of light–matter interactions to a greater extent.

Compared with single-layer metasurfaces, cascaded metasurfaces exhibit certain characteristics in terms of performance, fabrication, and cost. Performance-wise, cascaded metasurfaces offer enhanced functionality due to near-field coupling effects, demonstrate unavailable capabilities for single-layer devices, and present tunability employing dynamic configurations. At the same time, they are compatible with semiconductor processes for micro–nano-fabrication like single-layer metasurfaces [122,123], enabling low-cost batch production. Specific designs requiring double-sided processing on a single substrate may increase the manufacturing difficulty and cost moderately. This makes cascaded metasurfaces a promising option for widespread applications.

For future endeavors, machine learning algorithms may play crucial roles in complicated cascaded metasurface design, which has been observed in many works. For example, Fan et al. utilized the twisted diffractive neural network (TDNN) to boost the holographic storage capacity of meta-disks [108]. Bao et al. applied gradient descent for Jones matrix optimization in bilayer metasurfaces [94]. Georgi et al. achieved optical secret sharing through iterative gradient optimization of cascaded metasurfaces [33]. Wei et al. exploited an iterative scheme to unlock rotational multiplexing by leveraging in-plane rotation [26]. Moving forward, more efforts should focus on devising more potent algorithms integrating diverse techniques for refined structure–function tailoring. Interdisciplinary fusion with quantum and bio-optics holds promise for pioneering applications. Moreover, enhancing algorithm interpretability to clarify design physics is imperative. These strides will propel metasurface technology, opening vistas for optical innovation and heralding a new epoch in photonics research and application. 

## Data Availability

Data sharing is not applicable as no new data were created or analyzed in this paper.
